# Cluster FLISA—A Method to Compare Protein Expression Efficiency Between Cell Lines and Subunit Clustering of Proteins

**DOI:** 10.21769/BioProtoc.5484

**Published:** 2025-11-05

**Authors:** Sabrina Brockmöller, Lara Maria Molitor

**Affiliations:** Bundeswehr Institute of Pharmacology and Toxicology, 80937 Munich, Bavaria, Germany

**Keywords:** Cluster FLISA, Subunit clustering, Antibody amount detection, Protein expression

## Abstract

Nowadays, recombinant proteins are the focus of various research fields, and their use ranges from therapeutic investigations to cellular model systems for the development of therapeutic approaches. Cell systems used for the expression of recombinant proteins should be comparable in terms of yield and expression efficiency. In many research fields, it is desirable to obtain high protein concentrations. A method that combines an easy workflow with rapid results and affordable costs remains missing, and a standardized approach to determining protein concentration in transgenic cell lines is essential for more reliable data analysis. Our protocol demonstrates the cluster fluorescence-linked immunosorbent assay (FLISA), a technique that allows the exact quantification of comparable protein expression amounts. Moreover, it enables the detection of clustered or bound subunits of a protein without necessitating ultracentrifugation. In the present protocol, we demonstrate the utilization of two transgene cell lines, each expressing distinct recombinant proteins, to provide comparability of protein yields and detectable subunit clustering.

Key features

• Fast and cost-effective approach to integrate into laboratory practice.

• Different transgene cell lines are comparable regarding their transgene protein expression yields.

• Detection of clustering for up to four different subunits.

• Detection of precise antibody concentration for every protein subunit.

## Graphical overview


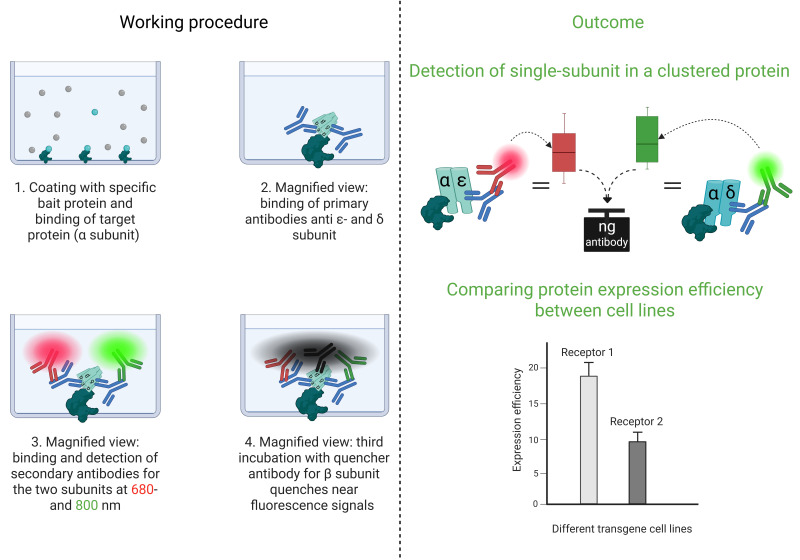


**Cluster FLISA procedure and its outcomes**

## Background

Recombinant cellular model systems are the method of choice for the development of therapeutic approaches in several research laboratories. The generation of numerous transgene cell lines allows for obtaining the most suitable model system for experimental investigations. It is necessary that the cell lines developed exhibit a range of characteristics that can be documented in the results, thereby ensuring the robustness of the data. To this end, we have devised a novel method, the cluster fluorescence-linked immunosorbent assay (cluster FLISA), to systemically validate model systems. We investigated various subtypes of the nicotinic acetylcholine receptor (nAChR), which are exemplarily used in this protocol. In particular, a comparison was made between generated [1,2] and purchased transgene cell lines, in order to determine which cell lines possessed the optimal characteristics for the experimental requirements. The characteristics examined included cellular host viability, the amount of transgene protein, and function. Data comparability was critical to selecting the right cellular system.

**Figure 1.**
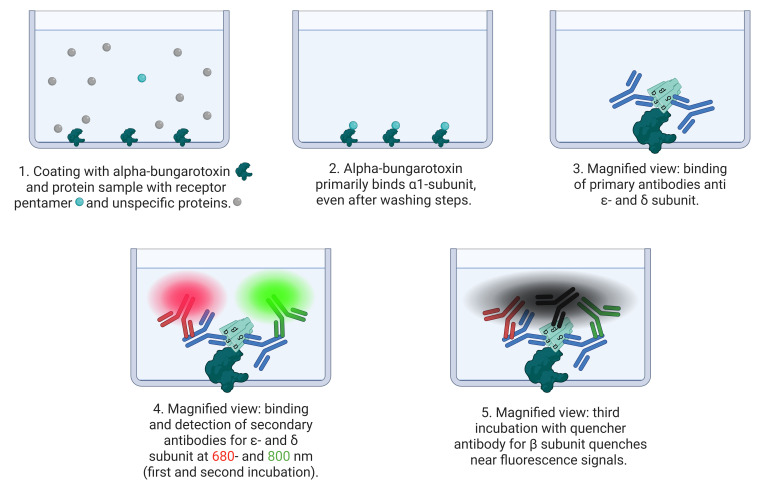
Principle of cluster FLISA assay. The scheme shows the process of cluster FLISA. 1. Coating the 96 microplate wells with the bait protein alpha-bungarotoxin and the protein sample solution. 2. Alpha-bungarotoxin binds receptor pentamers at the α1 subunit and washes out the unspecific protein sample. 3. In different runs, primary antibodies bind to ε- and δ subunits. 4. Subsequent detection by fluorescence-labeled antibodies at 680 and 800 nm. This enables the detection of different individual subunits. 5. In the final run of the cluster FLISA, a quencher-labeled antibody directed against the β1 subunit quenches nearby fluorescent signals. The tightly bound β1 subunit in the target protein is necessary for fluorescence signal quenching.

The cluster FLISA technique is similar to the known ELISA (enzyme-linked immunosorbent assay) method. The user employs a coated bait protein that binds the target protein [3] and an antibody to determine the concentration [4]. The cluster FLISA method represents an advancement over this method. The procedure is shown in Figure 1. It enables further antibody binding, specifically for each individual subunit of the target protein. By binding to one specific subunit, the bait protein enables the identification of all other subunits in the target complex via their associations. In the case of nAChR cluster determination, it is alpha-bungarotoxin that binds specifically to the α subunit [5–7]. If an additional subunit is detected, both are subjected to clustering or binding. This performance is executed in sequential steps that ensure the individual detection of each target subunit. The use of an antibody labeled with QC-1 quencher allows subsequent investigation of subunit clustering by extinguishing the detected fluorescence signals [8]. Moreover, it enables the use of a detection device with a limited number of fluorescence channels. By normalizing the protein concentration and analyzing the bound antibody concentrations, data can be compared across various cell lines and diverse proteins. The bait protein we used for the positive control of the assay and concentration determination of the bound antibodies was alpha-bungarotoxin conjugated to biotin. Other bait proteins could be made available by determining other target proteins. In addition, cluster FLISA enables the detection of subunit clustering of a protein without the need for ultracentrifugation, which represents a costly investment in the acquisition phase. Analytical ultracentrifugation is a method for determining the size, density, and shape of proteins [9].

The concentration of different transgene subunits was determined by measuring the amount of bound antibody. The clustering of the same subunit into a pentameric receptor was also analyzed. Both outcomes provided valuable insights into the quality of the model system. For instance, a transgene cellular model system with a good score for subunit clustering, but a decreased protein concentration, requires improved processing. We hypothesize that the maximum potential of recombinant cellular model systems will be attained by attempting to generate a state of comparable quality. The cluster FLISA is an easy tool for assessing these aspects, and it may be of interest to other research groups to qualify their models.

## Materials and reagents

In order to enhance the performance of cluster FLISA, it is necessary for the operator to possess purified transgene proteins. Please note that there are a couple of methods for protein purification, and we do not impose performance requirements for diverse protein columns or magnetic bead-based techniques. However, we recommend determining the protein concentration after the purification procedure to apply similar amounts. The purification of the protein solution used in this protocol was performed as described in our previous publications [1,2]. The subsequent protocol data exemplifies the utilization of cluster FLISA. It describes the exemplary proteins α1_2_β1δε nAChR and α7 nAChR, both of which have a pentameric structure. The α1_2_β1δε nAChR consists of four different subunits in a pentamer, whereas the α7 nAChR is a five-subunit homomeric structured protein. Both transgene cell lines expressing these proteins were generated in our laboratory [1,2]. Operators are required to modify antibodies and bait proteins to their target protein/subunits. Furthermore, we performed our cluster FLISA using the Odyssey CLx (LiCor) for plate scanning, but other devices are also possible for fluorescence scanning. The Odyssey CLx has two detection channels (680 and 800 nm wavelengths), which demonstrate how to use this device or others without scanning channels for every subunit. Any fluorescence scanning device can be used for analysis. Furthermore, the order of subunit detection is not specifically defined and can be freely selected.

**Biological materials**

Two recombinant Chinese hamster ovary (CHO) cell lines (Leibniz Institute DSMZ, ACC 110) stably expressing the α1_2_β1δε nAChR and the α7 nAChR.

*Note: The α1_2_β1δε nAChR consists of genetic tags His(6)-tag for α1, HA-tag for β1, Myc-tag for δ, and FLAG-tag for ε subunit, which was used for unambiguous identical detection.*

**Reagents**

1. ELISA phosphate coating buffer, 10 mM phosphate buffer, pH 7.4 (Invitrogen, Life Technologies, catalog number: CB07100)

2. Blocking buffer (LiCor, catalog number: 927-70001)

3. Bait protein Alpha-bungarotoxin conjugates (Invitrogen, Life Technologies, catalog number: B1601)

*Note: The bait protein is target-defined.*

4. Bait protein for positive control and concentration determination Alpha-bungarotoxin conjugates Biotin-XX (Invitrogen, Life Technologies, catalog number: B1196)

*Note: The bait protein is target-specific.*

5. PBS pH 7.4, without Mg^2+^ and Ca^2+^ (Gibco, catalog number: 10010-015)

6. Tween 20 (Merck/Sigma-Aldrich, catalog number: P1379-100mL)

7. Primary antibodies: His-tag (Abcam, catalog number: ab18184), HA-tag (Abcam, catalog number: ab49969), myc-tag (Abcam, catalog number: ab32), flag-tag (Abcam, catalog number: ab236777), and α7 subunit (Abcam, catalog number: ab216485)

8. Secondary antibodies: IRDye 800 rabbit (LiCor, catalog number: 92632211), IRDye 800 mouse (LiCor, catalog number: 92632210), IRDye 680 rabbit (LiCor, catalog number: 92668071), IRDye 680 mouse (LiCor, catalog number: 92668070), and IRDye 800 streptavidin antibody (LiCor, catalog number: 92632230)

9. Alexa Fluor dye 680 (Invitrogen, Life Technologies, catalog number: A20188)

*Note: Alexa Fluor dye 680 is no longer available; the successor product is Alexa Fluor dye 680, catalog number: A88069. The procedure remains the same.*

10. QC-1 quencher dye (LiCor, catalog number: 92970030)

11. Zeba spin desalting columns 7K MWCO, 75 μL (Invitrogen, Life Technologies, catalog number: 89878)

12. Bovine serum albumin (BSA) (Merck/Sigma-Aldrich, catalog number: A7906-50G)

13. Ultra-pure distilled water DNase/RNase-free (Invitrogen by Life Technologies, catalog number: 10977-035)

14. PBST (0.1% Tween-20 in PBS)

15. Primary antibody dilution 1:200 in blocking buffer

*Note: Antibody dilution was performed according to the manufacturer's instructions for use with ICC/IF.*

16. Secondary antibody dilution 1:1,000 in blocking buffer

*Note: Antibody dilution was performed according to the manufacturer's instructions for use with WB.*

**Solutions**

1. Solutions for cluster FLISA and antibody labeling process (see Recipes)

**Recipes**

**1. Solutions for cluster FLISA and antibody labeling process**

**Table:** 

Reagent	Final concentration	Notes
Alpha-bungarotoxin coating buffer	20 μg/mL	For a sample concentration of ≤ 0.1 mg/mL
Alpha-bungarotoxin Biotin-XX coating buffer	20 μg/mL	For a sample concentration of ≤ 0.1 mg/mL
QC-1 solution	4 μg/μL	The QC-1 dye is based on an NHS-ester formulation, which is unstable at the moment of solvation.
PBS-BSA solution	100 mg/mL	Fresh solution

**Laboratory supplies**

1. Black 96-well microplates with clear bottom (Greiner BioOne, catalog number: 655986)

2. pH indicator strips 6.5–10 (Meck, catalog number: 1095430001)

3. Protein LoBind^®^ tube 0.5 mL (Eppendorf, catalog number: 0030108094)

4. Protein LoBind^®^ tube 1.5 mL (Eppendorf, catalog number: 0030108116)

5. Protein LoBind^®^ tube 2 mL (Eppendorf, catalog number: 0030108132)

6. Eppendorf tube screw cap 5 mL (Eppendorf, catalog number: 0030122313)

## Equipment

1. Odyssey CLx Imager (LiCor, catalog number: 9140)

*Note: Odyssey CLx is no longer available; the successor model is Odyssey DLx Imager, catalog number: 9142.*

2. KS 260 control IKA (Sigma-Aldrich, catalog number: Z341835)

3. Fume hood HERA Safe KSP (Thermo Scientific, catalog number: 17168075)

4. Nanodrop 8000 spectrophotometer (Thermo Scientific, model: ND-8000-GL)

5. Mini Spin plus (Eppendorf, catalog number: 5453000011)

6. Rotina 420R (Hettich, catalog number: 4706SET2)

7. Thermo Mixer C (Eppendorf, catalog number: 5382000015)

8. Varioshake VA 15 T (Lauda, catalog number: LDL003059)

## Software and datasets

1. Image Studio software (version 5.2 LiCor)

2. Prism [version 9.5.1 (733) GraphPad]

3. Excel (version 16.0.10415.20025, Microsoft Excel 2019 MSO)

4. RStudio (version 2024.09.1 Posit Software, PBC)

5. R (version 4.4.2 R Resources)

## Procedure

**A. Antibody-labeling procedure**

*Note: The Odyssey CLx Imager has only two detection channels; the user needs a third detection option with an antibody labeled with a quencher dye to extinguish the fluorescence signals from the 680 and 800 nm channels. Furthermore, the QC-1 quencher dye alone is not detectable; in addition to calculating the labeling degree, the user must label another antibody with a visible dye as a control for labeling accuracy. In this example, the visible dye was Alexa Fluor dye 680.*

1. HA-tag ab49969 is the antibody in this example, which was labeled. For labeling, antibodies require a concentration of 1 mg/mL in a glycine-free stock solution.

*Note: Most antibodies are purchased at a maximum concentration of 1 mg/mL; if the stocking buffer contains glycine, the antibody must be dialyzed. This will result in a loss of antibody concentration, and the labeling procedure will not work below 1 mg/mL. Caution: We recommend purchasing a labeled antibody or selecting another one for the labeling process to ensure a successful procedure.*

2. The antibody has to be cleared of BSA or other peptides from the stocking buffer and replaced with PBS solution.

a. Perform Zeba spin desalting columns following the manufacturer’s protocol. Briefly, two columns are centrifuged at 1,500× *g* for 1 min in a Mini Spin Plus centrifuge to remove the column storage solution.

b. Wash the column resin three times with 300 μL of PBS and centrifuge at 1,500× *g* for 1 min.

3. Add the antibody solution to the center of the resin column bed and centrifuge at 1,500× *g* for 2 min. The collection tube should be a 1.5 mL protein LoBind^®^ tube.

4. Determine the concentration of purified antibody using the Nanodrop 8000 spectrophotometer in the *Protein 280* menu.

*Note: Some antibody solutions have concentrations up to 1 mg/mL; maintain the solution exactly to 1 mg/mL for best labeling efficiency.*

5. For labeling, purified antibody solutions have to be diluted 10:1 with 1 M sodium bicarbonate to adjust to a pH of 8.5. Check this value with pH-indicator strips with 0.5 μL per strip.

6. Add 100 μL of 1 mg/mL purified antibody solution to a vial of solid Alexa Fluor dye 680 from the labeling kit and resolve carefully. Incubate the solution protected from light at 20 °C in the Thermo Mixer C for 1 h.

7. For the second labeling procedure, dissolve the QC-1 dye in 125 μL of ultrapure water to obtain a concentration of 4 μg/μL. Vortex the solution for 10 s and rapidly add 2.49 μL of the QC-1 solution to 100 μL of purified antibody solution.

*Note: The QC-1 dye is based on an NHS-ester formulation, which is unstable at the moment of solvation.*

**Critical step:** Use QC-1 within 2–3 h after solvation to ensure labeling efficiency. Incubate protected from light at 20 °C in the Thermo Mixer C for 2 h.

a. The 2.49 μL of QC-1 dye solution is a variable volume and depends on the volume of antibody solution that has been labeled. In the following, we demonstrate the calculation for an example of a 100 μL purified antibody solution:

i. 100 μL of purified solution + 10 μL of 1 M sodium bicarbonate = 110 μL sample.

ii. The QC-1 dye manufacturer’s volume value is based on 1,000 μL of protein solution. The factor for 100 μL is 10 (see step A7a.v).

iii. Calculate the volume of QC-1 using its molecular weight of 1243.7 [g/mol]/150 [kDa] (antibody) = 8.291 μL.

*Note: The conversion of the result to μL is given by the manufacturer.*

iv. Calculate the volume value by the factor = 8.291 μL/10 = 0.8291 μL.

v. The QC-1 dye should be used at a dilution of 1:3, which is 0.8291 μL × 3 = 2.49 μL.

8. Resuspend both labeling reactions every 15 min.

9. After incubation, free dye particles must be removed from the labeled antibody solutions. Therefore, use purification resin in spin columns according to the manufacturer’s protocol for Alexa Fluor dye 680.

a. Add purification resin to the column according to the manufacturer’s protocol in a 5 mL Eppendorf tube with screw cap and centrifuge at 1,100× *g* for 10 s in a Rotina 420R centrifuge.

b. Repeat step A9a until the column is filled with purification resin.

c. Pack the column by centrifugation at 1,100× *g* for 4 min in a Rotina 420R centrifuge.

10. Add the two labeled antibody solutions to different purification columns, place each in a collection tube, and centrifuge at 1,100× *g* for 5 min in a Rotina 420R centrifuge.

11. Transfer the elution of both antibody solutions back to the appropriate column and repeat the centrifugation from step A10. Transfer the second elution to a 0.5 mL protein LoBind^®^ tube.

12. Determine the concentration of the purified labeled antibody solution in technical triplicates on the Nanodrop 8000 spectrophotometer using the *Protein 280* menu. Values are needed to determine the mean and correspond to the *Mean of A_280_* required in equation 1.

$${Antibody}_{concentration}=\frac{Mean\;of\;A_{280}-(Mean\;of\;A_{dye}\;\times \;correction\;factor)}{2030000}$$ (1)

13. Determine the absorbance for the degree of labeling in technical triplicates on the Nanodrop 8000 spectrophotometer using the *UV-VIS* menu. Use settings for the first absorbance of 280 nm and the second absorbance of *λ_max_* 679 nm (680 dye) or 750 nm (QC-1 dye). Values are needed to determine the mean and correspond to the *Mean of A_dye_* required in equations 1 and 2.

$$Degree\;of\;labeling=\frac{Mean\;of\;A_{dye}}{\varepsilon_{dye}\;\times \;result\;equation\;1}$$ (2)

14. Add 1 μL of 100 mg/mL BSA stock solution to the antibody solution if the concentration is less than 1 mg/mL.

*Note: The BSA concentration was chosen to be as high as described to avoid dilution of the antibody concentration solution.*

15. Control the pH to 7.2 with pH-indicator strips and store labeled, purified antibodies protected from light at 4 °C.

16. Calculate the labeling success for both labeled dyes. The correction factors *λ_max_* and *ε_dye_* were specified by the manufacturer for the 680 dye and QC-1 dye. When using Nanodrop, the user must use 2030000 in equation 1 [antibody concentration] and *ε_dye_* must be multiplied by 10, as stated by the manufacturer.

17. The range of the result of Equation 2, which indicates successful antibody labeling, is specified by the manufacturer for the 680 dye and QC-1 dye.

18. Calculate the volume of diluted antibody solution for cluster FLISA application. A primary antibody solution is needed in a dilution of 1:200, but the labeled antibodies are decreased in concentration by the purification procedure. Thus, the concentration value in step A12 must be multiplied by 200 to use the labeled antibody solutions in the assay.

**B. Cluster FLISA**

**B1. Plate coating with alpha-bungarotoxin**

1. Prepare alpha-bungarotoxin and alpha-bungarotoxin Biotin-XX coating buffer solutions separately to a final concentration of 20 μg/mL.

2. Add both alpha-bungarotoxin coating buffer solutions to a black 96-well microplate with 100 μL per well.

a. Alpha-bungarotoxin coating buffer solution must be added to columns 1A, B, F–H, 5–11A–H, and 12A–C, G, and H.

b. Alpha-bungarotoxin Biotin-XX coating buffer solution must be added to columns 2–4A–H.

**Figure 2.**
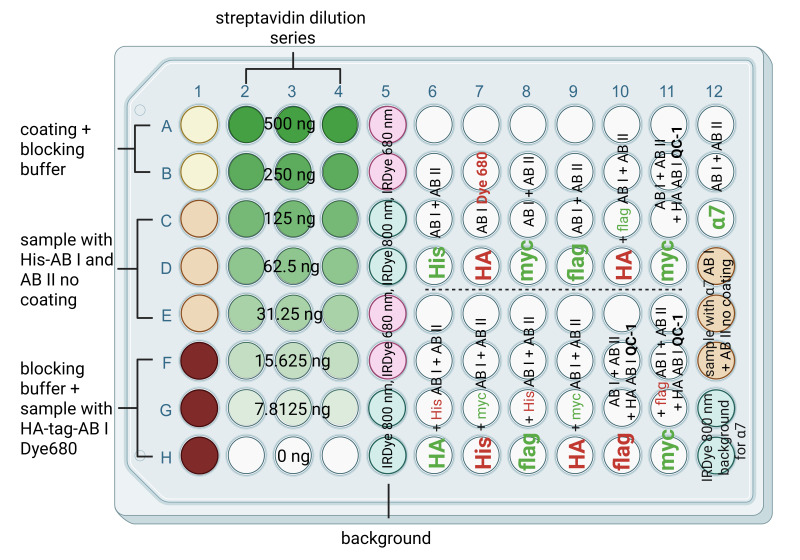
Schematic microplate construction for cluster FLISA assay. The scheme shows a 96-well microplate with column 1A–B containing negative controls for coating without sample, 1C–E and 12D–F containing negative controls for the sample without coating, 1F–H containing background for antibody labeling with visible dye (coating, no sample, and labeled antibody), and antibody dilution series 2–4 A–H for linear dilution of streptavidin solution to calculate detected fluorescence signals to antibody concentrations. The signals from columns 2–4 serve as a positive control for the successful binding of the bait protein. Furthermore, the successful binding and detection of streptavidin can be used as an example of successful antibody binding. Columns 5A–H and 12G, H contain coated wells and protein samples loaded with each secondary antibody solution to determine the background. Column 7A–D serves as an additional positive control for the successful labeling and detection of a particular signal by antibodies. Columns 6–12 contain the samples for protein detection. In detail, 6/7/8/9A–D and 12A–C show the detection of one subunit in the first run. 6/7/8/9E–H and 10A–D show the detection of two subunits in the first and second runs. Columns 10E–H and 11A–D each showed one subunit with fluorescent antibody detection and one subunit with QC-1 quencher detection. This further verifies that the QC-1 dye individually masks the fluorescence signals at 680 and 800 nm. Column 11E–H contains two subunits with fluorescent antibody detection in the first and second runs and one subunit with QC-1 quencher detection in the third run. For clarity, subunits detected in the first run are in bold; subunits detected in the second run are not in bold. In the third and final run, only the antibody labeled with quencher-QC-1 was applied. The red and green colored letters indicate which channel was detected in the respective run. The term AB I indicates primary antibody, AB II indicates secondary antibody, and QC-1 indicates quencher.

See Figure 2 for loading details.

**Pause point:** Incubate coated microplate overnight at 4 °C on a Varioshake at 50 rpm.

**B2. First run of cluster FLISA**

1. The next day, wash each well for 5 min twice with 150 μL of 0.1% PBST and once with 150 μL of PBS.

2. Block each well for 1 h at room temperature with 150 μL of blocking buffer at 150 rpm on a KS 260 control IKA.

3. Add 100 μL per well of the purified sample solution of α1_2_β1δε or α7 nAChR at a concentration of ≤0.1 mg/mL.

*Note: Solubilization and purification of both protein solutions were performed as reported in [1].*

4. For the binding process of the α subunit of the purified nAChR to alpha-bungarotoxin, incubate for 1 h at room temperature and 150 rpm on the KS 260 control IKA.

5. Repeat the washing procedure from step B2.1 and perform the first cluster FLISA antibody incubation with 100 μL/well of primary antibody dilution for 30 min at room temperature and 150 rpm on the KS 260 control IKA. See Figure 2 and Table 1 for loading details.

**Table 1.** Detailed microplate schedule for loading antibody dilutions in the respective runs of cluster FLISA

Run of cluster FLISA	Microplate well	Primary antibody dilution	Secondary antibody dilution
First	1 C–E, 6 A–D, 7 E–H	His-tag	
	6 E–H, 9 E–H, 10 A–D	HA-tag	
	1 F–H, 7 A–D	HA-tag 680 dye-labeled	
	8 A–D, 11 A–H	myc-tag	
	8 E–H, 9 A–D, 10 E–H	flag-tag	
	12 A–F	α7 subunit	
	1 C–E, 5 G–H, 6 A–H, 8 A–D, 11 A–H		IRDye 800 mouse
	5 E–F, 7 E–H, 9 E–H, 10 A–D		IRDye 680 mouse
	5 C–D, 8 E–H, 9 A–D, 12 A–H		IRDye 800 rabbit
	5 A–B, 10 E–H		IRDye 680 rabbit
	2–4 A–G		IRDye 800 streptavidin dilution
Second	6 E–H, 8 E–H	His-tag	
	7 E–H, 9 E–H	myc-tag	
	10 A–D, 11 E–H	flag-tag	
	6 E–H, 8 E–H		IRDye 680 mouse
	11 E–H		IRDye 680 rabbit
	10 A–D		IRDye 800 rabbit
	7 E–H, 9 E–H		IRDye 800 mouse
Third	10 E–H, 11 A–H	HA-tag QC-1 dye-labeled	

6. Repeat the washing procedure from step B2.1 and add 100 μL/well of secondary antibody dilution. Perform a dilution series for IRDye 800 streptavidin with a linear decrease of 500, 250, 125, 62.5, 31.25, 15.625, 7.8125, and 0 ng. Incubate for 1 h at room temperature, protected from light, at 150 rpm on the KS 260 control IKA. See Figure 2 and Table 1 for loading details.

7. Repeat the washing procedure from step B2.1 and add 100 μL/well of PBS prior to plate scanning with the Odyssey CLx. Use Image Studio software with the following settings: resolution = 169 μm, quality = medium, and focus offset = 3.8 mm.

*Note: Focus offset needs to be adjusted for different plates.*

8. To analyze expressed protein levels and clustered subunits of detected fluorescence signals, export the data as an Excel report.

**B3. Second run of cluster FLISA**

9. Perform the second cluster FLISA incubation with 100 μL/well of primary antibody dilution for 30 min at room temperature and 150 rpm on the KS 260 control IKA. See Figure 2 and Table 1 for loading details.

10. Repeat the washing procedure from step B2.1 and add 100 μL/well of secondary antibody dilution for 1 h at room temperature, protected from light, at 150 rpm on the KS 260 control IKA. See Figure 2 and Table 1 for loading details.

11. Repeat the washing procedure from step B2.1 and add 100 μL/well PBS prior to plate scanning with the Odyssey CLx. Use Image Studio software with the following settings: resolution = 169 μm, quality = medium, and focus offset = 3.8 mm.

*Note: Focus offset needs to be adjusted for different plates.*

12. Export the data as an Excel report.

**B4. Third run of cluster FLISA**

13. Perform the third cluster FLISA incubation with 100 μL/well of primary antibody dilution for 30 min at room temperature and 150 rpm on the KS 260 control IKA. See Figure 2 and Table 1 for loading details.

14. Repeat the washing procedure from step B2.1 and add 100 μL/well of PBS prior to plate scanning with the Odyssey CLx. Use Image Studio software with the following settings: resolution = 169 μm, quality = medium, and focus offset = 3.8 mm.

*Note: Focus offset needs to be adjusted for different plates.*

15. Export the data as an Excel report.

**B5. Additional required negative controls**

Additional negative controls are required and must be performed in a separate 96-well microplate. Therefore, sections B1, B2, B3, and B4 should be repeated with purified protein solution from host CHO cells that do not express the transgene protein. If the cluster FLISA was performed for cell lines that naturally express the target proteins, a protein solution without the target protein is required for the negative control.

## Data analysis

1. Raw data from all three runs need to be analyzed in GraphPad Prism or equivalent calculation software, such as Excel or RStudio. Figure 2 displays various controls that we recommend for data analysis. The negative controls (columns 1A–E and 12D–F, Figure 2) and the multiple backgrounds should detect signals that are not as strong as the detected sample signals.

*Note: Further negative controls from step B5 with alterations of purified samples of untransduced CHO cells without transgene proteins are needed. All detected signals should not be as high as those from protein samples from transgene cell lines.*

The positive controls indicate successful antibody labeling with a visible dye (column 7A–D, Figure 2), whereas the antibody dilution series detects fluorescence signals, demonstrating how the signal should be if bait (alpha-bungarotoxin) and prey (α subunit of nAChR) protein are bound (columns 2–4A–H, Figure 2). The technical triplicates of each streptavidin dilution were used to calculate the mean and determine the regression for quantitative translation of fluorescence units (FU) to antibody concentration. Each individual background signal (column 1F–H, 5A–H, and 12G–H, Figure 2) was recommended to average the secondary antibody fluorescence intensity. To analyze the data, each raw value had to be subtracted from the respective background mean (Equation 3).

2. Normalization of sample fluorescence intensities was performed using the protein sample concentration. Calculation of sample data was performed using Equation 3 and Equation 4.

equation 3=mean of sample FU-mean of respective background FU (3)

$$equation\;4=\frac{result\;of\;equation\;3}{slope\;of\;linear\;regression\;from\;streptavidin\;dilution\;series}$$ (4)

**Figure 3.**
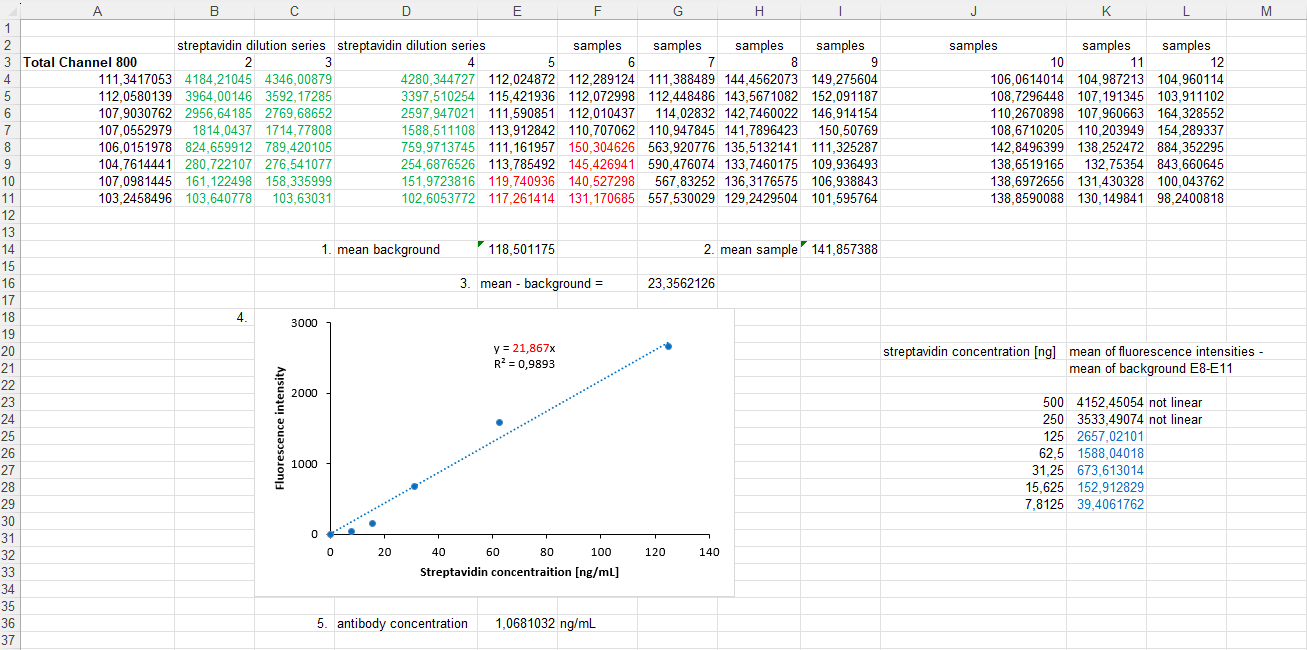
Example calculation of Equations 3 and 4 in an Excel sheet. Rows 6–12 show measured example data of a 96-well microplate. Column E consists of background signals; the examples are highlighted in red. Column F consists of sample signals; the examples are also highlighted in red. Below the raw data: 1. Calculation of the background signals' mean. 2. Calculation of the sample signal's mean. 3. Both results are needed to calculate the normalized fluorescence intensity by subtracting the background fluorescence intensity from the sample fluorescence intensity. (This calculation step is explained in Equation 3 of the protocol.) 4. The fluorescence intensities of the streptavidin dilution are used to generate a linear regression. The values used in the calculation are highlighted in green in columns B–D. The calculation of the mean and subtraction of the background are performed using the same steps as described in 1, 2, and 3. For clarity, the mean streptavidin intensities with the background subtracted are shown on the right side of the graph and are highlighted in blue. Subsequently, the linear regression was used to determine the slope, which is highlighted in red in the graph. 5. Calculate the antibody concentration of the sample by dividing the result from point 3 of this figure by the slope of the linear regression. (This calculation step is explained in Equation 4 of the protocol.)

The implementation of Equations 3 and 4 is further explained in Figure 3. The processed data from the fluorescence signals are used to create graph models such as boxplots or bar charts. We previously published our data in positive and negative bars to demonstrate the quenching of fluorescence signals by QC-1-labeled antibody [1]. We also declared on every bar chart the antibody concentration. Additionally, we performed a two-sample t-test with dependent samples with statistically significant p-values below 0.05.

3. Results of three runs of the cluster FLISA: The cluster FLISA procedure reveals diverse results. On the first run in our example of a pentameric protein, every single subunit was detected. The α subunit is the only one that binds specifically to alpha-bungarotoxin, meaning that if there is a detection of the β/δ/ε subunit, it can be concluded that they are clustered to the α subunit. To ensure unambiguous detection, it was necessary to cluster these three subunits with the α subunit. Furthermore, the individual detection of the β subunit (column 7A–D) serves as a control for the efficacy of antibody labeling and functional application in the cluster FLISA. The QC-1 dye is not detectable; it only quenches fluorescence signals. Therefore, the application of the method using visible dyes leads to improved process control.

The second run of cluster FLISA reveals further subunit clustering of the pentamer structure. Columns 6/7/8E–H detect the α subunit in conjugation with β/δ/ε, thereby demonstrating that antibodies themselves are not impeded by steric arrangement. Columns 9E–H and 10A–D indicate the clustering of subunits of β/δ and β/ε, facilitated by antibody detection and indirect α subunit bait-binding, respectively. Without the alpha-bungarotoxin bait-binding, no subunit of the pentamer nAChR is detectable by antibodies. The ability to detect individual subunits enables further in-process corrections, which can effectively eliminate false negative results caused by steric antibody hindrance.

The third run utilizes an antibody labeled with QC-1 dye to quench existing fluorescence signals with bound secondary antibodies from the preceding run. QC-1 has been described for a broad range of fluorescence signals. The data of columns 10E–H and 11A–D serve as controls for quenching 680 nm and 800 nm individuals. From these quenching results, further conclusions can be drawn for the clustering of β/ε and β/δ by antibody detection and, in both cases, indirectly by α subunit bait-binding. Complete quenching of the fluorescence signal by the QC-1 can be achieved by a tight binding of 65 Å [8] of the subunit signals to be quenched. Column 11E–H presents the comprehensive antibody detection results for δ (first run green fluorescence signal, Figure 2), ε (second run red fluorescence signal, Figure 2), β (third run fluorescence signal quenched by QC-1 dye), and α subunit by bait-binding (without bait-binding, no δ/ε/β subunit detection was possible).

4. Compare protein expression efficiency: The structural design of cluster FLISA facilitates single-subunit detection as well. The α7 nAChR, a homomeric pentamer, is detected during the initial run. The calculation of the fluorescence signal, as outlined in Equations 3 and 4, is essential for this analysis. The resultant value is then divided by 5 to determine the signal for a single α7 subunit. The subunits are then comparable to the mean of all single detected values of α1_2_β1δε nAChR. It is known from the literature that the expression efficiency of α7 nAChR is about 30% [10]. The efficiency for α1_2_β1δε nAChR is expected to be lower due to the increased complexity of the pentameric structure [10–14], but it has never been determined. The results of cluster FLISA allow, for the first time, a determination of expression efficiency for both receptors with detected values. The normalized comparability of the expression efficiency of different cell lines allowed conclusions to be drawn about the quality of the transgene cellular model system regarding recombinant protein yields, as well as data on subunit clustering.

## Validation of protocol

**Figure 4.**
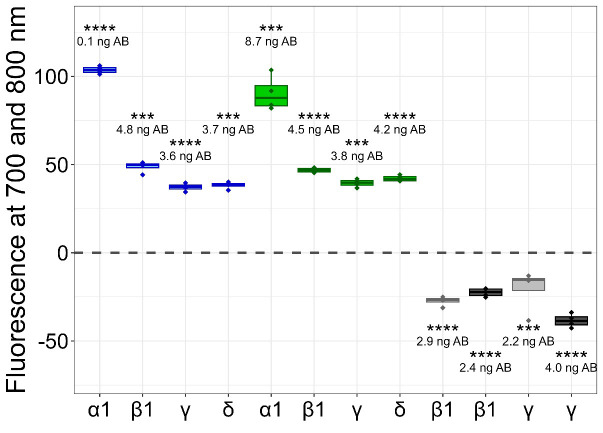
Detected fluorescence signals of clustered subunits for α1_2_β1γδ nicotinic acetylcholine receptor (nAChR). Detections of the first run are indicated by blue boxplots, detections of the second run are indicated by green boxplots, and detections of the third run are indicated by grey boxplots for individual α1/β1/γ/δ subunits. The light-grey boxplots show the quenching of one subunit, whereas the dark-grey boxplots show the quenching of two subunits. The fluorescence levels of subunits were derived from antibody (AB) concentrations, and their significance was compared to the respective backgrounds (first and second runs) or covered (third run) fluorescence signals. P-values below 0.001 were set as significant and marked with ***, and p-values below 0.0001 were also set as significant and marked with ****. The figure was created with RStudio (version 4.4.2).

This protocol or parts of it have been used and validated in the following research article(s):

• Brockmöller and Molitor et al. [1]. N-Glycosylation Deficiency in Transgene α7 nAChR and RIC3 Expressing CHO Cells Without NACHO. *The Journal of Membrane Biology* 257(3) (Figure 4, Method “cluster FLISA” and Results “Comparison of Expression of α7 and α1_2_β1δε nAChR Subtypes”).

This protocol has been applied to our laboratory practices for quality assessment between our multiple transgene cell lines, but these data are needed for further publications. For validation demonstration, in this protocol, we show the application of the cluster FLISA procedure to α1_2_β1γδ nAChR, which is also a pentameric receptor consisting of four different subunits. We purchased the purified protein from Cube Biotech (catalog number: 28603) and used an accurate protein concentration of 1.7 μg/well. We performed sections B1–4 of this protocol with the following changes: primary antibodies for target subunits that are not genetically tagged: ab288434 for α1, ab236959 for β1, ab233758 for δ, and ab151627 for γ. All these antibodies were purchased from Abcam. The antibody ab233758 was selected for labeling with 680 dye and QC-1 dye, and Figure 4 depicts reproducible results from the cluster FLISA for the α1_2_β1γδ nAChR.

## General notes and troubleshooting

**General notes**

1. The application of cluster FLISA in laboratory practice is complex in the first runs. We recommend a well-developed plan as an overview tool to prevent improper utilization of the sample or antibody solutions in the relevant wells.

2. The antibody labeling process requires an antibody concentration of 1 mg/mL and a glycine-free stock solution. To prevent the waste of funds due to unsuccessful antibody labeling or an insufficient degree of antibody labeling and labeling kit expenses, it is recommended to strictly verify the required properties of antibodies.

**Troubleshooting**

**Problem 1:** Insufficient detection: The bait protein concentration should be adjusted to match the protein sample solution concentration. For adequate working conditions, 10 μg/mL coating concentrations per well are sufficient for sample solutions of ≤0.1 mg/μL. Protein samples at lower concentrations are not detectable, indicating inefficient expression of complex proteins.

Possible cause: The low-concentration protein sample is below the detection minimum, resulting in false negative results.

Solution: For samples with a concentration ≥0.1 mg/μL, a bait protein coating of 20 μg/mL is required to detect fluorescent signals. We recommend the application values of Table 2.

**Table 2.** Values of bait protein concentration

Sample solution concentration	Bait protein concentration
≤0.1 mg/μL	10 μg/mL
≥0.1 mg/μL	20 μg/mL

**Problem 2:** Insufficient quenching: The subunits may be too far apart, resulting in insufficient quenching.

Possible cause: The clustered protein is very large, and the distance between the subunits exceeds 65 Å.

Solution: A user-selectable range for the degree of quenching is acceptable. We recommend no more than 5%.
